# Facilitating mental health research for patients, clinicians and researchers: a mixed-method study

**DOI:** 10.1136/bmjopen-2016-011127

**Published:** 2016-08-08

**Authors:** D Robotham, S Waterman, S Oduola, C Papoulias, T Craig, T Wykes

**Affiliations:** 1 Institute of Psychiatry, Psychology & Neuroscience, King's College London, London, UK; 2 South London and Maudsley NHS Foundation Trust, London, UK

**Keywords:** MENTAL HEALTH, research register, gatekeeping, recruitment, participation

## Abstract

**Objectives:**

Research registers using Consent for Contact (C4C) can facilitate recruitment into mental health research studies, allowing investigators to contact patients based on clinical records information. We investigated whether such a register was useful for mental health research, seeking the perspectives of patients and research investigators.

**Setting and design:**

In 2012, a C4C register was developed in a large secondary mental health provider within the UK; almost 9000 patients have joined. This mixed-method study audited the effectiveness of the register.

**Participants:**

A ‘mystery shopper’ exercise was conducted, and patients (n=21) were recruited to ask clinicians about the availability of research opportunities. Structured interviews were conducted with patients (n=52) about their experiences of being on the register. Similar interviews were conducted with 18 investigators from 19 studies, who had attempted to use the register to recruit participants.

**Outcome measures:**

The impact of C4C on study recruitment, and whether it helped patients learn about research.

**Results:**

So far, the register has provided 928 individuals with 1085 research opportunities (in 60% of cases, the individual agreed to participate in the study). Clinicians were willing to link patients to research opportunities, but often lacked information about studies. For patients, the register provided opportunities which they may not otherwise have; 27 of 52 had participated in studies since joining the register (18 participating for the first time). Most investigators used the register to supplement recruitment to their studies, but described problems in prescreening potential participants from a clinical record for complex studies.

**Conclusions:**

Although the register helped investigators recruit for studies, and provided patients with research opportunities, clinicians' input is still useful for identifying suitable participants. C4C registers should be adapted to provide clinicians with automatically updated information on local studies allowing them to match patients on their caseload with active studies.

**Strengths and limitations of this study**

- Describes how electronic health records can be used to facilitate research.
- Mixed-method study (including mystery shopper exercise) evaluates the system in real-world settings.
- Highlights the importance of clinicians in the research recruitment process, and describes how to monitor and improve the system in order to account for this.
- Getting accurate recruitment statistics relies on the diligence of research investigators who use the register.

## Introduction

Mental health research is underfunded,^1^ but even funded research is affected by poor recruitment rates,^2^ while clinical gatekeeping^3^ reduces participation further and therefore hampers the chances of success. Developments in IT infrastructure and healthcare governance enable researchers to use patient clinical records as a basis for screening and contacting potential participants, if the patient has given permission.^4^ ^5^ These ‘Consent for Contact’ (C4C) research registers link patients to research opportunities without requiring clinicians to match patients to individual studies. Such a system may increase the number of research opportunities being offered to people with mental illness, and increase recruitment to studies in this field.

A C4C register was developed and implemented across South London and Maudsley NHS Foundation Trust (SLaM), a large secondary mental health provider. Information regarding the implementation of the register has been published elsewhere. Procedural, technical and information governance frameworks were developed with oversight from the NHS Trust's Caldicott Guardian,^5^ and local patients and clinicians were involved in the development of the register. Clinicians were provided with training and materials to help them explain the key concepts of the register to patients and to clarify concerns, such as patients' right to accept or decline invitations to participate in specific studies.^6^ The register was advertised to all Trust staff through banners, screensavers and in the induction programme for all new staff. A dedicated team was set up to promote C4C to clinicians, and to help them enrol people. This team has visited all clinical teams across the Trust.

A pilot study of the register's implementation found that patients welcomed autonomy in research participation, but expressed concerns about confidentiality and about the register possibly leading to coercion to participate in subsequent research studies.^7^ Following the findings of the pilot study, training materials were devised through which clinicians could present the register to their clients in a way that would enable informed consent and address their concerns where appropriate. The team charged with implementation of the register also provided specialist support to teams of high need, such as short-term funding to help staff nurses recruit people to the register.

At the time of writing, 12 370 patients have been asked whether they wished to join the register, with 8862 (71%) agreeing to join. Key demographic features of the register are 56% men, 90% below the age of 65, 58% were listed as ‘white’ ethnicity, 23% as ‘black’, 4% as ‘Asian’, 6% as ‘mixed’ and 11% as ‘other’. Enrolment to the register has been growing at an average rate of 258 per month since 2012. While the sign up rate is one measure of success, there is no information about how the register facilitates research, such as whether it improves recruitment rates and enables patients to learn about (and participate in) mental health research. This article fills these gaps by specifically assessing the impact a C4C register has on local research activity.

## Method

### Design

This was a mixed-method cross-sectional design. Data collection procedures were approved by the local NHS Trust audit committee.

Phase 1 involved collecting data about clinicians' and service providers' awareness of research. This was performed through a ‘mystery shopper’ exercise in which mental health outpatients from the Trust would ask their service provider a question about whether any research opportunities were available. Mystery shopping is an observational research method which is often used in consumer and marketing research as a way of gaining feedback about consumer experiences. Such a method has been used for similar purposes in the past.^8^

Phase 2 involved collecting feedback from stakeholders about their experience of the C4C register. Data were collected from (1) patients who had been registered on C4C and (2) investigators who had used the C4C to recruit participants for a study.

### Participants

#### Phase 1

Mystery shoppers were recruited through adverts in Trust hospitals, through a register of patients who were interested in ‘service user involvement’ opportunities, and through visits to local patient/service user organisations. The inclusion criterion was that they were currently using Trust outpatient services. A mixture of convenience, snowball and purposive sampling was used to recruit. The aim was to ensure a sample of shoppers who were attending different clinical service providers, in different areas across the NHS Trust. We aimed to recruit a total of 20 shoppers.

#### Phase 2a

Patients were recruited using the C4C register itself, and parents/carers were interviewed when the patient was below consenting age or lacked mental capacity. The inclusion criterion for this phase was that the patient had been offered at least one opportunity to participate in research via the register, that is, an investigator had previously contacted them and asked them whether they wanted to take part in a particular research project.

#### Phase 2b

Investigators were contacted through email invites. We identified all those who had submitted application forms to use the register for recruitment. The inclusion criteria were that investigators had begun recruiting for the project and had obtained the necessary governance approvals. In some cases, principal investigators provided alternative contacts for members of their research team who had conducted recruitment. An investigator from every study was recruited.

### Materials

All materials used were designed for the purpose of this study (copies are available under online supplementary information).

10.1136/bmjopen-2016-011127.supp1 Supplementary data

#### Phase 1

A researcher met with mystery shoppers, explained the aims of the exercise and gave them a list of example questions and prompts which could be used in discussions about research opportunities with a clinician. For example, ‘Do you know of any studies/trials coming up that I might participate in?’. Shoppers completed a Mystery Shopper Feedback Questionnaire after conducting their visit. This 14-item questionnaire asked about the outcome of the visit, including a mixture of yes/no, 5-point Likert scale (‘strongly agree’ to ‘strongly disagree’) and open-ended questions. It was designed for completion by hand or online.

#### Phase 2a

Participants completed a Patient Audit form. This consisted of yes/no and open-ended questions about access to research opportunities since joining the register. They were also asked about how they knew about the research register, their previous experience of research participation and 5-point Likert scale questions about their experiences of being on the register. A final open-ended question asked how the register could be improved.

#### Phase 2b

Investigator participants were asked to complete a Researcher Audit form. This covered the following: (1) recruitment—inclusion criteria, whether the register was a primary or secondary source of recruitment, how it worked for prescreening participants; (2) an overview of how the investigator had used the register—eight questions on a 10-point rating scale, relating to usefulness, ease of use, whether they would use it again and (3) comparison with other recruitment methods—six questions on a 5-point Likert scale which asked investigators to rate C4C as a recruitment method. The number of patients recruited to each study was available on the C4C recruitment database.

### Procedure

#### Phase 1

Each mystery shopper was trained in how to ask their service provider about research opportunities. In all cases, they decided to ‘shop’ at the service where they were receiving input. Shoppers questioned their service provider at the next appropriate consultation and did not reveal their mystery shopper status during the consultation. Shoppers chose either to meet with their clinician face to face or to conduct the exercise over the phone. After completing the exercise, shoppers returned the feedback questionnaire to the study team and were paid £15 for their time. The average time between shoppers receiving training and completing the task was 11 days; this ranged from one shopper completing the task on the same day to one completing 31 days later. This phase took place between November 2013 and December 2014.

#### Phase 2a

Structured interviews with patients (and carers where appropriate) were conducted via telephone between November 2014 and December 2015. Written notes were taken during the telephone call. The data were then entered into an SPSS Version 20 database.

#### Phase 2b

Structured interviews with researchers were conducted face to face. They were audio-recorded. The data were inputted into an SPSS database. Researchers were followed up after they had completed recruitment to their study to gather updated data on the number recruited from C4C. Interviews occurred between April 2014 and April 2015.

### Data analysis

SPSS descriptive statistics were used to analyse the quantitative data. Open-ended questions provided textual data; questions that elicited short textual responses were categorised and treated numerically in SPSS. A small amount of qualitative data was gained. These data were analysed thematically by two independent raters, and indicative quotes are presented.

## Results

### Sample characteristics

#### Phase 1

All mystery shoppers (n=21) were users of outpatient mental health services. More than half (n=13) had previous experience of research. Most had known the staff member they were approaching for <3 years (n=16). As outpatients, shoppers either approached community-based teams (n=15) or hospital-based teams (n=6). In total, 13 different clinical teams were visited. In most cases, the exercise was conducted in person (n=15), with the remaining six doing it by phone. Of the services visited by shoppers, three had received specialist input and training from the C4C programme team. In two cases, shoppers visited before this occurred, and the remaining shopper visited their team within 2 months after the team had received specialist support.

#### Phase 2a

A total of 52 patients were recruited: 29 men and 23 women. In seven cases, the parent or carer was interviewed instead of the patient. Participants' ages varied; four were using child and adolescent mental health services, seven were using older adults' mental health services (including dementia services) and the remainder were using adult mental health services. All participants had joined the C4C research register (or were speaking on behalf of someone who had joined it). All participants had previously been contacted by a research investigator who had attempted to recruit them for a study.

#### Phase 2b

Eighteen investigator participants were interviewed in relation to 19 studies. Investigator participants included 4 men and 14 women; they had a spread of recruitment experience ranging through 1–2 years (n=7), 3–4 years (n=4), 5–9 years (n=6) to 10+ years (n=1). The studies (n=19) covered a range of topics: dementia (n=4), psychosis (n=4), attention deficit hyperactivity disorder (n=1), personality disorder (n=1), depression (n=2) and obsessive compulsive disorder (n=2). A further five studies were not specific to any particular illness. One investigator was responsible for two different studies.

### Phase 1: shopping for research opportunities

All but one of the shoppers agreed that their queries about research opportunities had been understood by staff (yes=20, neutral=1). Few staff explicitly mentioned the research register to shoppers (yes=3, no=18), but almost half mentioned research projects which the shopper might participate in (yes=9, no=11, unsure=1). In most cases, shoppers reported that clinicians were helpful (yes=15, no=3, neutral=3). Over half reported that clinicians provided them with details about where to go for further information (yes=11, no=8, unsure=2). In some cases (n=5), shoppers reported that staff promised to look for relevant information and report back later, as exemplified in the quotes below:

Although [the nurse] was unaware about research she was not dismissive. She said she was not sure but she could try and find out the information needed. (Shopper #10)

Staff were willing to ask around and referred me to PALS [Patient Advice and Liaison Service]. (Shopper #15)

The nurse did not know much but said she would make phone calls and let me know. (Shopper #21)

### Phase 2a: patients' experiences of being on the research register

Of the 52 patients interviewed, 27 had participated in a study they had been contacted about. Only 15 reported ever taking part in research before joining the register. Of the remaining 37 patients, 18 had taken part in research for the first time since joining the register and 19 had yet to take part in any research. Direct contact with investigators was viewed as positive, allowing for greater choice and information about research. Over half (n=30) agreed that the register allowed them to choose what they wanted to participate in (only 1 disagreed, 12 gave a neutral answer and 9 did not know). Twenty-nine said that it allowed them to be more informed about research (12 gave a neutral answer, none disagreed, 11 did not know). None of the participants said that having direct contact with the researcher was a negative experience, most (n=29) said it was positive, 9 gave a neutral answer and 14 did not know. The register also appeared to facilitate the direct liaison between researchers and potential participants; in 35 cases, the patients were contacted first by the researcher and in 8 cases by the care team (the remaining 9 participants did not know).

Only seven people said there were things they disliked about being on the register: not feeling safe on the register or worrying about data safety and security (n=3), receiving too many calls about research (n=1), not wanting to take part in studies that were offered (n=1), finding the research studies confusing (n=1) or preferring not to be called at all (n=1). When asked about how the register could be improved, the most common request was that reminders should be provided (n=7):

Need more reminders about what it actually **is**. (Patient #18)

I want more information about it; I don't understand what it is. (Patient #24)

It wouldn't hurt to have a leaflet or some dialogue. (Patient #3)

### Phase 2b: investigators' opinions about using the register

In the 19 studies audited, investigators recruited 194 participants via the register (mean=10, SD=18.7). The three highest recruiting studies had recruited 73, 43 and 26 participants, respectively, from the register; three further studies had recruited 13, 11 and 10 participants. Six studies did not recruit any participants at all through this method. The register was the primary source of recruitment in only three studies. Other recruitment methods were used in all studies; examples of these methods included liaison/referrals with clinical team (n=19), other databases, networks and registers (n=6), advertising, leaflets and posters (n=4).

Eligibility criteria were a key variable as to whether C4C was useful. One limitation was that investigators needed to use the patient record to screen participant eligibility. The three highest recruiting studies had simple eligibility criteria, for example, recruiting people based on whether they had used a particular type of service, rather than whether they were taking specific medication/dose, or had a certain diagnostic profile. These studies varied in recruitment population, one recruiting children and young people, one recruiting older adults and one recruiting a general adult mental health sample. Two were observational studies and one was a multicentre trial. Nine investigators described how searching the register returned participants who later proved unsuitable because the record was incorrect or had become outdated: ‘we experienced a scenario several times whereby patients had stopped taking their medications so were no longer suitable for the study’. Investigators showed wariness in relying on the register to screen potential participants. They described the need for clinical judgement in recruitment:

There is no way of identifying whether the participants are in crisis and sometimes [contacting the clinician] is a better way of checking whether the [person] meets our exclusion criteria. (Investigator #10)

Quite a few [were] later found unsuitable when checking the notes. (Investigator #16)

Still needed a clinical eye over the notes for details. (Investigator #18)

Most investigators (n=14) thought using C4C was worthwhile, and all would recommend it to colleagues (n=18). The data in table 1 show the degree to which investigators valued C4C (higher scores indicating increased usefulness). Studies were categorised into three groups depending on their recruitment success using C4C: those recruiting 10 or more participants from C4C, those recruiting between 1 and 10 and who did not recruit any. Data show that studies failing to recruit any participants from C4C found it less valuable and useful than studies that managed to recruit. Studies in which 10 or more participants were reported finding C4C the most useful and reliable, and reported being most likely to use C4C again. Nonetheless, even those who did not recruit reported that they were likely to try it again.

**Table 1:** Investigators' opinions of Consent for Contact (C4C) in relation to their studies

	Total (n=19)	10+ participants recruited (n=6)	Between 1 and 9 participants recruited (n=7)	0 participants recruited (n=6)
C4C was valuable	5.5 (SD=2.9)	7.0 (SD=3.5)	5.9 (SD=2.1)	3.7 (SD=2.4)
C4C was useful	6.1 (SD=2.8)	7.8 (SD=1.5)	6.7 (SD=2.2)	3.5 (SD=2.7)
C4C was easy to use	7.1 (SD=1.5)	7.2 (SD=.8)	6.7 (SD=1.9)	7.5 (SD=1.5)
Likely to use C4C again	8.5 (SD=1.7)	9.7 (SD=.5)	8.4 (SD=2.1)	7.5 (SD=1.5)

Half of the investigators agreed that it was faster and more time effective than other recruitment rates (n=9), and six said that it helped recruit more participants than other methods (eg, posters and adverts, snowball sampling and clinical contacts).

## Discussion

A C4C register has the potential to facilitate mental health research, providing mental health patients with more research opportunities, and helping researching investigators to recruit participants for studies. By April 2016, there were 1085 research participation opportunities (to 928 patients) which investigators had inputted on the system (this is likely to be an underestimate of the real number of approaches, since some investigators may not have logged their usage of the register correctly). Of these approaches, there had been 654 recruits to studies (a 60% participation rate), from 538 individuals. Of this number, 63% were male, the age range was between 7 and 97 (mean=39, SD=22.6) and the ethnicity statistics were 46% white, 36% black, 4% Asian, 7% mixed and 7% other (or lacking information). This shows that women have been under-represented in recruitment, in comparison with their representation on the register itself. In contrast, black ethnic groups have been over-represented, a finding that could be seen in the context of research recruitment of black and minority ethnic groups.^9^

When asked, most patients agree to join the register; it has led to the recruitment of participants and provided opportunities for people who may never have participated in any research before. Findings highlight two factors that influence the effectiveness of the register: (1) the amount of information clinicians are given (or can recall) about local research opportunities and (2) the limitations of clinical records as a prescreening tool for investigators.

Clinicians are willing to help patients find out about research, but they may not be equipped to do so. Ongoing audit and monitoring should be performed in order to guide and improve the implementation of the register. Since this audit was conducted, feedback has been provided to service directors and information about the register was added to staff screensavers, clinical inductions and job descriptions. The purpose of this was to make the register more visible to clinical staff. Designated staff members were also made available to help teams enlist people onto the register. Clinicians' workloads mean that those working in mental health services often struggle to prioritise research alongside clinical work.^10^ This means that they may not remember to mention C4C when discussing research with patients. Therefore, information about research must be provided to clinicians in a way which complements their clinical work, and researchers can also make clinicians feel more involved in the research process, and ensure that their contribution is valued.

Investigators tended to use C4C as one of several recruitment methods. Unsurprisingly, those who successfully recruited participants reported finding it more useful than those who did not. Nonetheless, those investigators who did not recruit were keen to use the system again in future. The primary difficulty experienced by investigators lay in screening potential participants from the register; this became a problem when the studies had more stringent, subjective or time-limited eligibility criteria. Participants' present life circumstance is a common recruitment criterion in mental health research. The subtle information that is necessary to screen for eligibility may not be available in clinical records, as they may not always contain the latest information about patients.^11^ ^12^ This led to investigators relying on more traditional methods of recruitment. At present, investigators' ability to use the register might depend on the study eligibility criteria they have set, through which investigators might judge whether using C4C is likely to be worthwhile for their study.

The problems experienced by investigators in using the register might be solvable by extending the data linkages that underpin it. At present, clinicians cannot use their caseload management system to match patients on their caseload to active research projects. In future, automated, personalised prompts could potentially be made available to clinicians as part of their caseload management system. The clinician could then confirm patients' suitability based on the clinical knowledge of those on their caseload. This information could then be sent back to the researcher. This would provide clinicians with access to information about suitable research projects, and provide researchers with all important information about participant suitability. This would ensure that clinicians are updated about research, and can see automatically which projects might be relevant to which patients.

This study demonstrates the utility of C4C registers to increase recruitment and provide opportunities for seldom heard individuals to get involved in research. However, it also confirms the importance of clinicians in screening participants for mental health research studies. Clinical records are insufficient for screening potential participants for complex eligibility criteria which are often the norm in mental health research. This means that clinicians need to feed into the participant screening process for those complex studies and the next stage in efficient use of these registers must be to adapt them to provide clinician support for their use.

### Strengths and limitations

The strength of this study is that it took place within real-world settings. The mystery shopper method allowed for the collection of data which would not otherwise had been possible to collect through other means. Conducting follow-up interviews with shoppers might have strengthened this phase of the study, and revealed whether staff had provided shoppers with post-appointment information. The collection of recruitment statistics from C4C relied on researchers' diligence in logging their usage of the system and inputting it back into the clinical record, which may not always be the case. The data collection method may therefore underestimate the number of times people have been approached and recruited using the register.

## References

[1] Wykes T, Haro JM, Belli SR Mental health research priorities for Europe. Lancet Psychiatry 2015;2:1036–42. 10.1016/S2215-0366(15)00332-6 26404415

[2] Patterson S, Kramo K, Soteriou T The great divide: a qualitative investigation of factors influencing researcher access to potential randomised controlled trial participations in mental health settings. J Mental Health 2010;19:532–41. 10.3109/09638237.2010.520367 21121823

[3] Howard L, de Salis I, Tomlin Z Why is recruitment to trials difficult? An investigation into recruitment difficulties in an RCT of supported employment in patients with severe mental illness. Contemp Clin Trials 2009;30:40–6. 18718555 10.1016/j.cct.2008.07.007 PMC2626649

[4] Cheah S, O'Donoghue S, Daudt H Permission to contact (PTC)—a strategy to enhance patient engagement in translational research. Biopreserv Biobank 2013;11:245–52. 10.1089/bio.2013.0023 24845592

[5] Callard F, Broadbent M, Denis M Developing a new model for patient recruitment in mental health services: a cohort study using Electronic Health Records. BMJ Open 2014;4:e005654 10.1136/bmjopen-2014-005654 PMC4256538 25468503

[6] Robotham D, Riches S, Perdue I Consenting for contact? Linking electronic health records to a research register within psychosis services, a mixed method study. BMC Health Serv Res 2015;15:199 10.1186/s12913-015-0858-4 25971412 PMC4432971

[7] Papoulias C, Robotham D, Drake G Staff and service users’ views on a ‘Consent for Contact’ research register within psychosis services: a qualitative study. BMC Psychiatry 2014;14:377 10.1186/s12888-014-0377-6 25539869 PMC4296527

[8] Wilson AM The use of mystery shopping in the measurement of service delivery. Serv Ind J 1998;18:148–63. 10.1080/02642069800000037

[9] Chang TE, Brill CD, Traeger L Association of race, ethnicity and language with participation in mental health research among adult patients in primary care. J Immigr Minor Health 2015;17:1660–9. 10.1007/s10903-014-0130-8 25398517

[10] Borschmann R, Patterson S, Poovendran D Influences on recruitment to randomised controlled trials in mental health settings in England: a national cross-sectional survey of researchers working for the Mental Health Research Network. BMC Med Res Methodol 2014;14:23 10.1186/1471-2288-14-23 24533721 PMC3928923

[11] Sohel J, Clark BS, Paton C Allergies and adverse drug reactions: clinical records versus patients’ perceptions. J Mental Health 2009;18:51–6. 10.1080/09638230701530218

[12] Tsai J, Bond G A comparison of electronic records to paper records in mental health centers. Int J Qual Health Care 2008;20:136–43. 10.1093/intqhc/mzm064 18079127

