# Supplementary material for: Facilitating mental health research for patients, clinicians and researchers: a mixed-method study
Source: BMJ Open. 2016 Aug 8;6(8):e011127. doi: 10.1136/bmjopen-2016-011127 (PMC4985796; doi:10.1136/bmjopen-2016-011127)
Supplement: Supplementary data [file bmjopen-2016-011127supp.pdf]

Phase 1

INFORMATION ON RESEARCH OPPORTUNITIES IN SLaM – AN EVALUATION

FEEDBACK QUESTIONNAIRE

1. Which South London and Maudsley service did you contact? (This information is for eligibility purposes only and will not be reported in our findings)

2. How did you contact the SLaM service?

In Person ☐

By Phone ☐

Other (please specify)

3. Did staff mention research projects you could take part in?

☐ Yes ☐ No ☐ Not sure

4. Did staff talk to you about research registers, 'consent for contact', C4C, or something similar to this?

☐ Yes ☐ No ☐ Not sure

5. Did staff tell you where you could get more information (about research) from?

☐ Yes ☐ No ☐ Not sure

6. How did you feel when talking to staff about research participation?(Please tick all that apply)

- Confident ☐
- Anxious ☐
- Concerned ☐
- Embarrassed ☐
- Excited ☐
- Confused ☐
- Curious ☐
- Fearful ☐
- Angry ☐
- Neutral ☐
- Other (please specify)

For Questions 7-9, please tick one answer to indicate how much you agree with the following statements:

7. Staff understood my questions about research

☐	☐	☐	☐	☐
Strongly Agree	Agree	Neither Agree nor Disagree	Disagree	Strongly Disagree

8. Staff were helpful to me in answering my question(s) about research

☐	☐	☐	☐	☐
Strongly Agree	Agree	Neither Agree nor Disagree	Disagree	Strongly Disagree

9. Staff were well informed about research in the Trust

☐	☐	☐	☐	☐

Strongly Agree	Agree	Neither Agree nor Disagree	Disagree	Strongly Disagree

10. What other comments do you have about your conversation?

11. Have you been involved in research before? If so, what types?

12. Have you signed up to any research projects since?

13. Have you signed up to a research register (such as C4C / ‘consent for contact’) since speaking to staff about research?

☐ Yes ☐ No

14. How long have you known the member of staff you spoke with?

THANK YOU FOR YOUR PARTICIPATION!

Phase 2a

C4C AUDIT FORM

Date:

Background Information

**CAG**	☐ M<br>☐ F	☐ SU<br>☐ Proxy
**Age group** ☐ under 18<br>☐ 19-29<br>☐ 30-39		

Contact with Researcher

Have you been contacted by a researcher recently?	
Did you end up participating in the project?	
Did anyone from your care team contact you before the researcher did?	
Had you participated in research projects before registering with C4C?	
Was there anything you disliked about being contacted through the register?	
Was there anything you liked about being contacted this way?	

Assessment of C4C in operation

	Strongly Disagree	Disagree	Neither	Agree	Strongly Agree

**Dealing with the researcher directly was a positive experience**	☐	☐	☐	☐	☐
Comments					
**Being on C4C allows me to choose which projects I want to participate in**	☐	☐	☐	☐	☐
Comments					
**Being on C4C allows me to be more informed about research**	☐	☐	☐	☐	☐
Comments					

Final Evaluation

How do you think C4C could be improved? (think about what you liked and what you disliked about your experience)	

Phase 2b

C4C AUDIT FORM

Date:

Background Information							
**Job Title**		**Band**	☐ M	☐ KCL	☐ Other	☐ Principle Investigator	☐ Other
**Role** (briefly list key responsibilities)							
**Years of Recruitment Exp.**	☐ 1-2 ☐ 3-	**Which of the following recruitment methods have you used in the past?** ☐ Referral ☐ Advertising			**Are you still using C4C?** ☐ Yes	**When did you you use C4C?**	
**Project Description** (type, length, phases, question)							

Recruitment	
What were your recruitment criteria? (age, diagnosis, ethnicity, location, medication, etc)	
Are you still recruiting?	
Would you be able to provide recruitment statistics for your project?	
What percentage of participants was recruited from C4C?	
Were you given any training or assistance in using C4C? If so, what was it?	
Was C4C the primary method of recruitment?	
What other methods did you use?	

Did C4C work in pre-screening participants?	
Did anyone later prove unsuitable?	
Did you meet your recruitment target?	

C4C

How did you find out about C4C?	

Overview of the Experience

SCALE: 1 (not at all) to 10 (very [illegible]	1	2	3	4	5	6	7	8	9	10
**How valuable was C4C to your project?**	☐	☐	☐	☐	☐	☐	☐	☐	☐	☐
Comments										
**How easy was it to use?**	☐	☐	☐	☐	☐	☐	☐	☐	☐	☐
Comments										
**How likely are you to use C4C again?**	☐	☐	☐	☐	☐	☐	☐	☐	☐	☐
Comments										
**Would you use it again for a similar project?**	☐	☐	☐	☐	☐	☐	☐	☐	☐	☐
Comments										
**I received enough information about how to use C4C.**	☐	☐	☐	☐	☐	☐	☐	☐	☐	☐
Comments										
**How reasonable was it to obtain access to the system?**	☐	☐	☐	☐	☐	☐	☐	☐	☐	☐
Comments										
**How reasonable was it to maintain access to the system?**	☐	☐	☐	☐	☐	☐	☐	☐	☐	☐
Comments										
**How useful was C4C to you?**	☐	☐	☐	☐	☐	☐	☐	☐	☐	☐
Comments										

Compared to other recruitment methods C4C

	Strongly Disagree	Disagree	Neither	Agree	Strongly Agree
**produced more participants who matched recruitment criteria**	☐	☐	☐	☐	☐
Comments					
**was faster**	☐	☐	☐	☐	☐
Comments					
**was more time-effective**	☐	☐	☐	☐	☐
Comments					
**was worthwhile**	☐	☐	☐	☐	☐
Comments					
**required a lot of effort**	☐	☐	☐	☐	☐
Comments					
**is one I would recommend to a colleague**	☐	☐	☐	☐	☐
Comments					

Final Evaluation

How could C4C be improved?	
